# On invariant T cells and measles: A theory of “innate immune amnesia”

**DOI:** 10.1371/journal.ppat.1009071

**Published:** 2020-12-17

**Authors:** S. M. Mansour Haeryfar

**Affiliations:** 1 Department of Microbiology and Immunology, Western University, London, Ontario, Canada; 2 Department of Medicine, Division of Clinical Immunology & Allergy, Western University, London, Ontario, Canada; 3 Department of Surgery, Division of General Surgery, Western University, London, Ontario, Canada; 4 Centre for Human Immunology, Western University, London, Ontario, Canada; University of Michigan Medical School, UNITED STATES

Measles (aka rubeola) is a highly contagious disease caused by a single-stranded RNA virus belonging to the family Paramyxoviridae. Measles virus (MeV) caused major epidemics and claimed an estimated 2.6 million lives annually prior to 1963 when a measles vaccine was introduced. Immunization against measles is estimated to have prevented 23.2 million deaths between 2000 and 2018. Notwithstanding, MeV continues to ravage impoverished regions, especially in the aftermath of natural disasters. In addition, despite the availability of safe, effective and affordable vaccines against MeV, anti-vaccination sentiments have created vulnerable societal pockets, which can be troubling in light of the increasing ease of global travel. It is therefore not surprising that recent years have witnessed a multitude of measles outbreaks and that measles-associated deaths have been alarmingly on the rise.

## Measles-induced immunosuppression

Many deaths from measles are due to secondary infections with unrelated pathogens amid a profound state of immunosuppression [1,2]. The three receptors dictating the cellular tropism of MeV are CD46 (aka membrane cofactor protein) [3,4], nectin 4 (aka poliovirus receptor-related 4) [5,6], and CD150 (aka signaling lymphocyte activation molecule family member 1) [7–9]. Wild-type (WT), laboratory-adapted and vaccine strains of MeV utilize or hijack CD150 to interact with or infect permissive immunocytes, and measles-elicited lymphopenia and antigen (Ag)-presenting cell (APC) impairments have been linked to CD150 expression.

The acquired immune deficiency of patients with measles was reported as early as in 1908 when a failure to mount normal tuberculin skin reactions was observed in children following measles [10]. The mechanisms underlying measles-associated immunosuppression, which can last for weeks to months, are wide-ranging and far from fully understood. Macrophages and dendritic cells (DCs) become infected with MeV and serve as Trojan horses transferring the virions to select lymphocyte subpopulations [11–13]. Exposure to or infection with MeV alters signal transduction pathways operating in DCs and interferes with their survival, maturation, and pro-inflammatory cytokine production and naïve T cell priming capacities [14–16]. T/B cell lymphopenia, retarded T cell proliferation in response to nonspecific mitogens [17], a prolonged cytokine imbalance in favor of T helper 2 (T_H_2)-type and anti-inflammatory mediators [e.g., interleukin (IL)-4, IL-13 and IL-10] [18,19], and CD4^+^CD25^+^ regulatory T cell accumulation in the peripheral blood [20] are among other immunological consequences of measles.

## Adaptive immune amnesia

**Fig 1:**
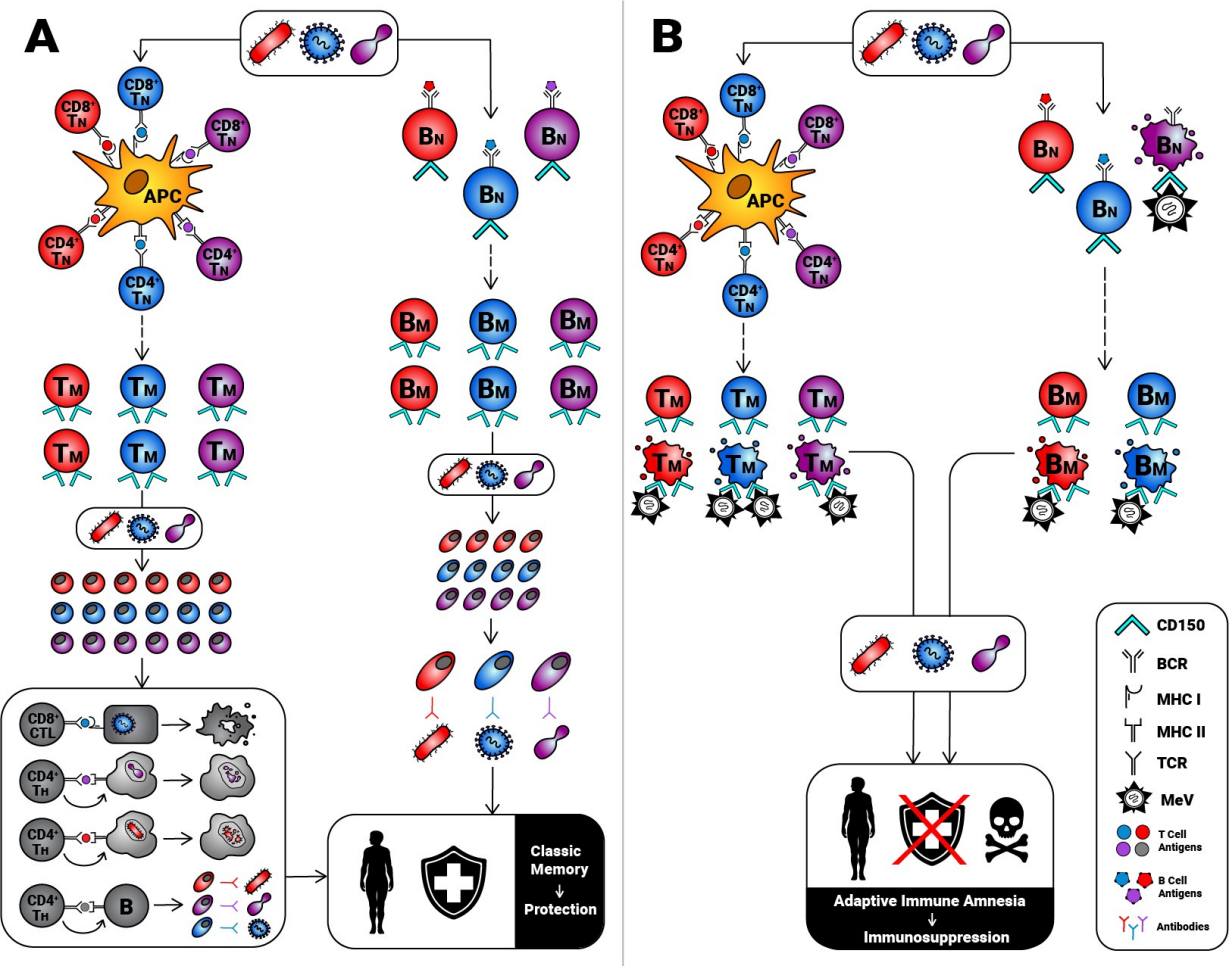
By killing memory cells, MeV causes adaptive immune amnesia. (A) Primary T and B cell responses to various microbial pathogens generate diverse memory cell pools. Once reexposed to cognate antigens, memory cells respond specifically, quickly and strongly to eliminate pathogens through cell-mediated and humoral mechanisms that are often protective. (B) MeV binds CD150 and destroys naïve and memory B cells as well as memory T cells. As a result, memory cell repertoires are depleted of many specificities, and a state of adaptive immune amnesia is established, which is responsible, at least partially, for measles-associated immunosuppression and heightened susceptibility to infections that are unrelated to measles. APC, antigen-presenting cell; BCR, B cell receptor; B_M_, memory B [cell]; B_N_, naïve B [cell]; CTL, cytotoxic T lymphocyte; MeV, measles virus; MHC, major histocompatibility complex; TCR, T cell receptor; T_H_, helper T [cell]; T_M_, memory T [cell]; T_N_, naïve T [cell].

By infecting and eliminating preexisting memory cells that express high levels of CD150, MeV erases the recollection of past exposures to microbes and “resets” the host’s defense system back to its default. This is referred to as “immune amnesia” [21], which is thought to contribute to measles immunosuppression (Fig 1A and 1B).

Using human tonsillar tissue blocks, Condack and colleagues reported that WT MeV strains infect approximately 5 to 6 times more CD3^+^CD45RO^+^ memory T (T_M_) cells than CD3^+^CD62L^+^CD45RA^+^ naïve T (T_N_) cells [22]. These results were recapitulated in an in vivo setting in which intratracheal infection of rhesus macaques and cynomolgus macaques resulted in the preferential infection of CD4^+^ and CD8^+^ T_M_ cells [23]. de Vries and colleagues subsequently demonstrated that MeV infects central memory T (T_CM_) and effector memory T (T_EM_) cells present in the peripheral blood, tracheobronchial lymph nodes, tonsils/adenoids, and Peyer’s patches of infected macaques [21]. Importantly, a numerical decline in the T_M_ compartment was followed by massive lymphoproliferation. Accordingly, the authors posited that measles lymphopenia, driven by T_M_ depletion, can be masked by the expansion of MeV-specific lymphocytes and bystander cells. This may explain, at least partially, the sustained immunosuppression of measles patients despite their seemingly short-term lymphopenia.

Unlike their naïve counterparts, human blood and tonsillar T_CM_ and T_EM_ cells are highly susceptible to MeV infection in vitro [21,24,25]. By comparison, naïve B (B_N_) and memory B (B_M_) cells are comparable in their permissiveness to MeV [24]. A 2013 measles outbreak that afflicted unvaccinated children from a Dutch Orthodox Protestant community provided Laksono and coworkers with a unique opportunity to analyze serial peripheral blood mononuclear cell (PBMC) samples collected before and after measles [26]. These investigators confirmed T/B lymphopenia during prodromal measles. They also detected MeV-infected CD4^+^ and CD8^+^ T_M_ cells, but not T_N_ cells, which had reached their peak frequencies approximately 1 day before the onset of the characteristic measles rash. By contrast, infected B_N_ and B_M_ cells were detectable at similar levels. Importantly, comparing pre- and post-measles samples revealed significant drops in IgG^+^ and IgA^+^ B_M_ cell percentages [26]. Although B_M_ cells are not the only B cell population targeted by MeV, their depletion should compromise recall responses of humoral nature to a plethora of pathogens. Consistent with this notion, experimental infection of rhesus macaques and natural infection of unvaccinated children with MeV shrunk the size of preexisting antibody repertoires by up to 60% and 73%, respectively [27], likely due to long-lived plasma cell (LLPC) deletion. Some of the missing specificities should recover upon reexposure to cognate Ags. However, some antibodies may be permanently lost if B_N_ and B_M_ cells, along with LLPCs, are drastically eliminated. In fact, B cell receptor (BCR) sequencing of pre- and post-measles PBMCs by Petrova and colleagues showed inadequate reconstitution of B_N_ cell pools as well as a loss of previously expanded B_M_ cell clones after MeV infection [28].

Petrova and colleagues also employed a ferret model of influenza A virus (IAV) vaccination to document the functional significance of immune amnesia [28]. Infecting IAV-vaccinated ferrets with canine distemper virus (CDV), a morbillivirus closely related to MeV, lowered IAV-neutralizing antibody titers, indicating the abolishment of “serological memory” otherwise maintained by plasma cells. Moreover, CDV infection curtailed the animals’ recall response to a secondary IAV challenge and worsened the severity of their flu symptoms.

## Unmasking the innate face of immune amnesia

Immunological memory is commonly viewed as a hallmark feature of adaptive immunity. However, numerous studies in the past decade or so have demonstrated that priming innate and innate-like immune cells with a pathogen may prepare or “train” them for stronger responses to the same, similar or even somewhat dissimilar microbes [29].

Up until recently, immune amnesia was discussed exclusively in the context of adaptive immunity, and whether MeV also targets innate, “memory-like” T cells remained unknown. These include mucosa-associated invariant T (MAIT) and invariant natural killer T (*i*NKT) cells, which are regarded as emergency responders to microbial invaders [30–33].

**Fig 2:**
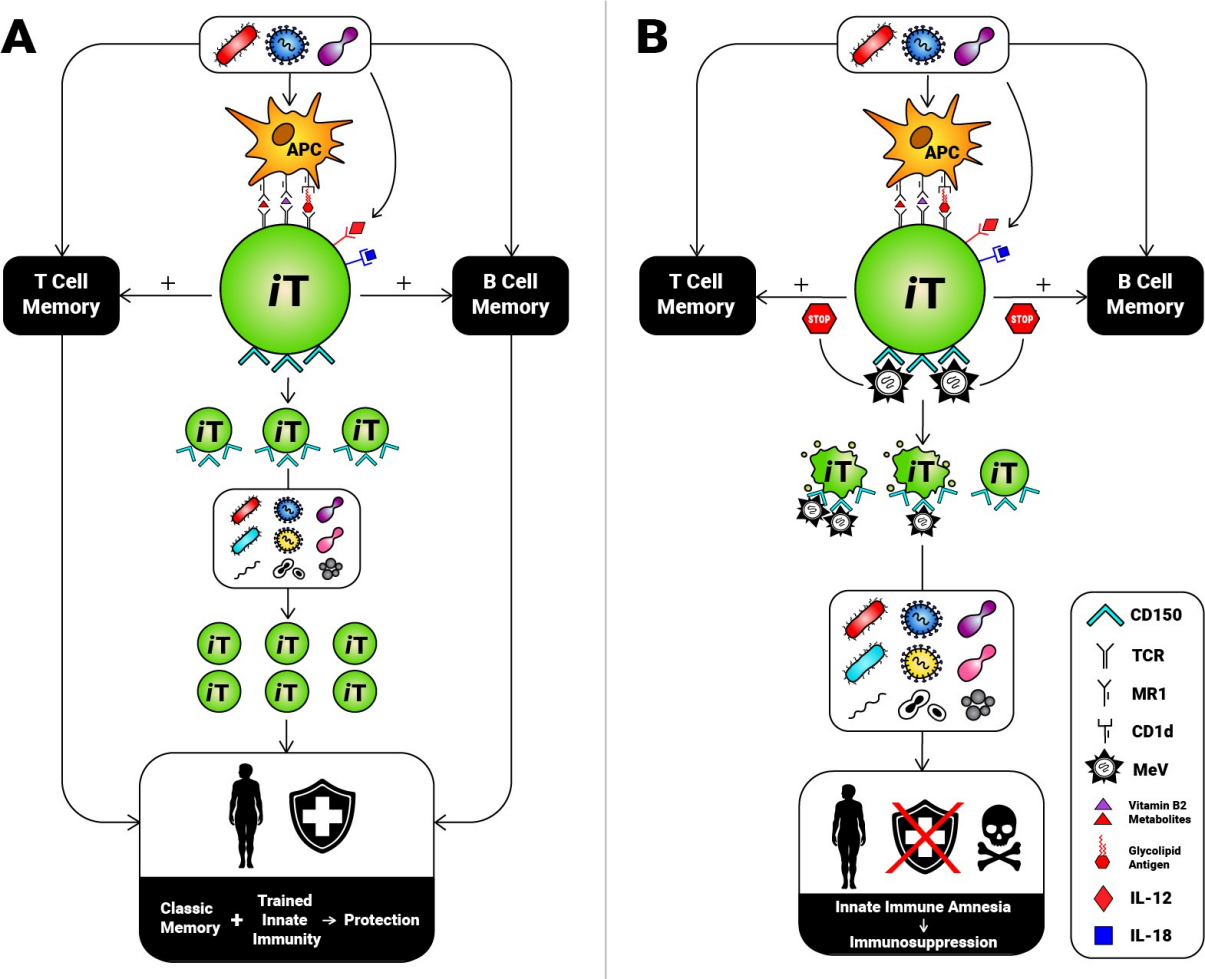
Invariant T cell depletion by MeV results in innate immune amnesia and contributes to adaptive immune amnesia. (A) MAIT and *i*NKT cells recognize certain bacterial and/or fungal components displayed by the monomorphic antigen-presenting molecules MR1 and CD1d, respectively. These *i*T cells can also be activated during viral infections in a cytokine-dependent manner, primarily through IL-12 and IL-18 receptor signaling. *i*T cells may remember their encounter with pathogens or may be “trained” to launch more robust responses to the same, similar, or unrelated microbes in the future. They also promote classic memory cell responses, thus indirectly aiding in cell-mediated and humoral immunity to cognate microbial antigens. (B) *i*T cells express very high levels of CD150 and may be consequently targeted and eliminated by MeV. The net effect is hindered trained immunity and compromised classic memory against a broad spectrum of pathogens, which together cause severe immunosuppression and increased proneness to many infections. APC, antigen-presenting cell; IL, interleukin; *i*NKT, invariant natural killer T [cell]; *i*T, invariant T [cell]; MAIT, mucosa-associated invariant T [cell]; MeV, measles virus; MR1, MHC-related protein 1; TCR, T cell receptor.

MAIT cells recognize riboflavin precursor derivatives of bacterial and fungal origin, among other compounds, in the context of MHC-related protein 1 (MR1) [34–37] (Fig 2A). *i*NKT cells detect glycolipid Ags, including those derived from pathogens, presented within the hydrophobic pocket of CD1d [38–40] (Fig 2A). The antimicrobial properties of these invariant T (*i*T) cells are owed to their tremendous immunomodulatory and cytolytic activities [32,41–43].

Several phenotypic markers of NK cells (e.g., CD56 and CD161) and mainstream T cells (e.g., CD3) and their memory subset (e.g., CD45RO) are also expressed by many *i*T cells [44–47]. Therefore, *i*T cells may have been given mistaken identities in previous studies, including but not limited to those conducted on measles. This is particularly important in the case of MAIT cells that form a substantial fraction of peripheral blood CD3^+^, CD8^+^, and CD45RO^+^ (or CD45RA^−^) cells in humans. Of note, human MAIT cells can be loosely divided into interferon (IFN)-γ-producing MAIT-1 and IL-17-producing MAIT-17 subsets with transcriptionally overlapping features [48,49]. Interestingly, of functional T cell subsets, T_H_17- and T_H_1/T_H_17-type cells are reportedly most sensitive to MeV infection in vitro [24] and circulate at reduced frequencies in children with measles [26]. The latter observation lends credence to the possibility of MAIT cell depletion during measles.

Using tetramer reagents to precisely identify human MAIT [36,50] and *i*NKT cells [51,52], we recently demonstrated that CD150 is more highly expressed by *i*T cells than by any other lymphocyte subsets, including T_CM_ and T_EM_ cells [25]. We found MeV to effectively infect *i*T cells and to quickly program MAIT cells for apoptotic death. Based on these results, it is plausible to assume that MeV tracks and destroys *i*T cells, thus putting an important component of innate antimicrobial defense out of commission. This, in turn, renders measles patients prone to a wide range of unrelated infections (Fig 2B). This phenomenon may be referred to as “innate immune amnesia” for several reasons. First, most *i*T cells have a T_EM_-like phenotype [45–47]. Second, genuine memory *i*T cell pools may exist [53–55]. Third, exposing MAIT cells to infectious agents or vaccine adjuvants may result in their clonal expansion and sustained activation and may also “train” them to respond more vigorously when they encounter the same agent, other stimuli, or even heterologous infections [56–59] (Fig 2A). This is distinct from the fine specificity of adaptive memory. Fourth and perhaps most definitively at this point, *i*T cells promote conventional memory cell responses [53,59–64]. As such, their depletion by MeV should also negatively impact classic immunological memory (Fig 2B).

The concept of innate immune amnesia is supported by additional findings. First, some of the bacterial culprits of post-measles infections are recognized and responded to by MAIT cells in vitro [65,66]. Second, reactivation of infection with *Mycobacterium tuberculosis*, which is known to elicit MAIT cell responses [55], has been reported after measles [67]. Third, measles-induced mortality due to immunosuppression is typically caused by secondary infections in the respiratory tract, MeV’s main port of entry, and also in the gut [68–70]. These are among locations in which MAIT cells strategically reside and operate.

## Implications and future directions

MAIT cells comprise up to approximately 10% of all T cells in the human peripheral blood and a substantial fraction of immune cells in mucosal layers—hence their denomination. They fulfill critical roles early in the course of antibacterial and antifungal host responses. They may detect and kill infected cells displaying MR1-bound Ags [42,71–74], transactivate other antipathogen effectors such as NK cells, *i*NKT cells, and cytotoxic T lymphocytes (CTLs) [75–77], and facilitate memory B cell responses [59,64]. They may also participate in tissue repair [78–81], thus indirectly assisting with the maintenance of tissue-resident memory compartments. Therefore, MAIT cell depletion should have serious repercussions for host defense.

The final extrathymic stage of MAIT cell maturation and their homeostasis in the periphery require the presence of commensal bacteria and B cells [82,83]. One may thus speculate that measles-induced peripheral B cell depletion may affect the numerical reconstitution of MAIT cells after measles. To this end, MAIT cells and B cells, among other MeV targets, need to be compared for their reconstitution kinetics. From a qualitative standpoint, it will be important to ascertain whether returning MAIT cells will exhibit bias, for instance in their cytokine production capacity, as they will be gradually reoccupying their niches. This may be studied longitudinally after MeV is cleared and no longer considered an existential threat to either MAIT cells or B cells.

Unlike in mice, *i*NKT cells are rare in the human peripheral blood and in human tissues. This is with the exception of the omentum [84], historically dubbed as the “abdominal policeman” [85], which serves to promote peritoneal immune responses [86]. Therefore, *i*NKT cell elimination by MeV may be detrimental to protective immunity in specialized sites.

Viruses are devoid of the riboflavin biosynthesis machinery that supplies prototypic MAIT Ags [35–37] and of glycolipids that stimulate *i*NKT cells. However, they can still activate *i*T cells in an MR1/CD1d-independent fashion, predominantly through cytokines like IL-12 and IL-18 [87–89] (Fig 2). As such, innate immune amnesia should also weaken host responses to viral pathogens other than MeV.

To conclude, *i*T cell depletion by MeV represents a novel immune evasion strategy that impedes memory responses to bacteria, yeasts, and viruses, either directly or indirectly. Therefore, MAIT and *i*NKT cells may be considered the missing link for measles immunosuppression at the interface between the innate and adaptive arms of immunity. Future studies on experimental and natural measles will need to document the contributions of innate amnesia to immunosuppression in the wake of measles.
